# Abnormal Macrophage Polarization in Patients with Myelodysplastic Syndrome

**DOI:** 10.1155/2021/9913382

**Published:** 2021-07-09

**Authors:** Gaochao Zhang, Liyan Yang, Yu Han, Haiyue Niu, Li Yan, Zonghong Shao, Limin Xing, Huaquan Wang

**Affiliations:** Department of Hematology, General Hospital, Tianjin Medical University, Tianjin, China

## Abstract

**Background:**

This study is aimed at assessing the subsets of bone marrow macrophages in patients with myelodysplastic syndrome (MDS) and exploring the role of macrophages in the pathogenesis of MDS.

**Methods:**

Thirty-eight newly diagnosed MDS patients were enrolled in the Department of Hematology of General Hospital of Tianjin Medical University from June 2015 to June 2016. Bone marrow monocytes and macrophage subsets (M1/M2) were detected in patients with MDS and normal controls by flow cytometry. M1 macrophages were cultured *in vitro*, and the expression of IL-1*β* and TNF-*α* mRNA was measured using real-time polymerase chain reaction.

**Results:**

Compared with the normal control group, the proportion of bone marrow monocytes was higher (2.11 ± 0.93% vs. 3.66 ± 3.38%), and the mean fluorescence intensity of surface molecule CD14 was lower in the higher-risk (HR) MDS group (639.05 ± 359.78 vs. 458.26 ± 306.72, *p* < 0.05). The ratio of M2 macrophages to monocytes was higher in patients with HR-MDS (1.82 ± 2.47% vs. 3.93 ± 3.81%, *p* < 0.05). The ratio of M1 to M2 macrophages was lower in the HR-MDS group (3.50 ± 3.22 vs. 1.80 ± 0.88, *p* < 0.05). The expression of IL-1*β* and TNF-*α* mRNA in M1 macrophages was significantly lower in the MDS group (*p* < 0.05).

**Conclusions:**

Patients with MDS had abnormal macrophage polarization, which may be involved in the alteration of bone marrow microenvironments.

## 1. Introduction

Myelodysplastic syndrome (MDS) is a heterogeneous group of malignant and clonogenic diseases that originate from hematopoietic stem cells. The main features are abnormal hematopoiesis (myeloid cell development abnormalities) and ineffective hematopoiesis (one line or multilineage). Approximately 30% of patients develop acute myeloid leukemia (AML) during the course of the disease. The pathogenesis of MDS is associated with genetic mutations, epigenetic changes, and bone marrow microenvironments [1, 2].

The monocyte macrophage system is mainly composed of monocytes and macrophages. Its main function is to remove pathogens or waste materials from the blood and tissues, and it also plays a key role in the induction and regulation of the adaptive immune response [3]. However, recent studies have suggested that monocytes and macrophages are part of the bone marrow microenvironment related to homing, mobilization, senescence of hematopoietic stem cells, and the formation of erythropoiesis [4]. Macrophages are differentiated from monocytes. The polarization of macrophages is usually divided into two categories: classical polarizing I macrophages (M1) and type II macrophages (M2) as a substitute for polarization [5]. Classically activated M1 polarized macrophages have antitumor activity and might induce tumor tissue destruction. Tumor progression is related to the transition from the M1 to M2 phenotype. In the late stage of tumor progression, macrophages usually have an M2 phenotype, with low IL-12 expression, high IL-10 expression, low tumoricidal activity, and promotion of tissue remodeling and angiogenesis [6].

Our previous studies showed that the number of monocytes in the peripheral blood of MDS patients increased, but the ability to differentiate into macrophages and the phagocytic function decreased [7]. The macrophages in the bone marrow are a part of the bone marrow microenvironment. Different macrophage polarization states play important roles in the differentiation of hematopoietic stem cells. We speculate that the M1/M2 polarization of bone marrow macrophages in patients with MDS may be an important factor in the pathogenesis and progression of MDS.

In this study, we evaluated M1 and M2 macrophages from the bone marrow of MDS and the culture of M1 macrophages *in vitro*.

## 2. Methods

### 2.1. Patient Characteristics

The MDS group enrolled 38 newly diagnosed MDS patients in the Department of Hematology of General Hospital of Tianjin Medical University from June 2015 to June 2016, including 20 males and 18 females, with a median age of 58 (range, 21-79) years. According to the International Prognostic Score System (IPSS), the patients were divided into the lower-risk (LR) MDS group (15 cases) and the higher-risk (HR) MDS group (23 cases) (detail in Table 1). The control group consisted of 21 healthy controls (11 males and 10 females) with a median age of 38 (23–65) years. This study was approved by the Ethics Committee of the General Hospital of Tianjin Medical University (IRB2021-WZ-052). Informed written consent was obtained from all patients and controls or their guardians according to the Helsinki Declaration.

### 2.2. Flow Cytometric Method

Bone marrow samples were obtained by standard bone marrow puncture using sterile heparin anticoagulant tubes. Bone marrow samples were filtered using flow cytometry tubes. CD14-FITC (Cat No.: 555397), CD68-PE (Cat No.: 565595), CD64-APC (Cat No.: 561189), CD40-PEcy7 (Cat No.: 561215), CD206-PE (Cat No.: 555954), CD163-PEcy7 (Cat No.: 556018), and isotype control antibodies (BD Biosciences, USA) were added to the tubes. The samples were then stained for 15 min in the dark at room temperature. After red blood cell lysis, the cells were washed with PBS. Finally, the cells were detected using a FACSCalibur flow cytometer (BD Biosciences, USA). Data analysis was performed using the Cell Quest software (Becton Dickinson, version 3.1).

Macrophages were defined as CD14^+^CD68^+^ cells. M1 macrophages were defined as CD64^+^CD40^+^ macrophages. M2 macrophages were defined as CD206^+^CD163^+^ macrophages (detail in Supplemental Figure 1).

### 2.3. M1 Macrophage Cell Culture In Vitro

Peripheral blood mononuclear cells (PBMCs) were separated from fresh heparinized blood samples (5 mL) using Ficoll Solution (Suolaibao, China). The PBMCs were seeded at 3 million cells/mL in sterile RPMI 1640 (Invitrogen, CA, USA) and cultured for 7 days with granulocyte-macrophage colony-stimulating factor (GM-CSF) (Huabei Pharmacy, Shijiazhuang, China), interferon-gamma (Sigma, USA), and lipopolysaccharide (Sigma, USA). On day 7, macrophages were collected from the bottom of the culture dishes.

### 2.4. Real-Time Polymerase Chain Reaction (qPCR)

Total RNA from macrophages was extracted using TRIzol (Takara Bio, CA, USA), and cDNA was generated using a reverse transcriptase kit (Takara Bio, CA, USA). Gene expression was quantified by qPCR (SYBR® Premix Ex Taq II, Takara Bio, China). The primer sequences were as follows: IL-1*β* forward 5′-GATCACTGAACTGCACGCTCC-3′ and reverse 5′-ACTTGTTGCTCCATATCCTGT-3′, TNF-alpha forward 5′-GGAGAAGGGTGACCGACTCA-3′ and reverse 5′-CTGCCCAGACTCGGCAA-3′, and GAPDH forward 5′-GCACCGTCAAGGCTGAGAAC-3′ and reverse 5′-TGGTGAAGACGCCAGTGGA-3′. The relative quantification (RQ) of gene expression was performed using the 2^-*ΔΔ*Ct^ method: ΔΔCt = (Ct_target_ − Ct_GAPDH_)_patients_ − (Ct_target_ − Ct_GAPDH_)_controls_.

### 2.5. Statistical Analysis

The results were analyzed using the GraphPad Prism 8.0 program (GraphPad Software, Inc., San Diego, CA). Data with normal distribution were presented as means ± SD, and multiple group comparisons were performed using one-way analysis of variance (ANOVA). Statistical significance was set at *p* < 0.05.

## 3. Results

### 3.1. The Quantity of Monocytes Was Increased in the Bone Marrow of Patients with HR-MDS

**Figure 1:**
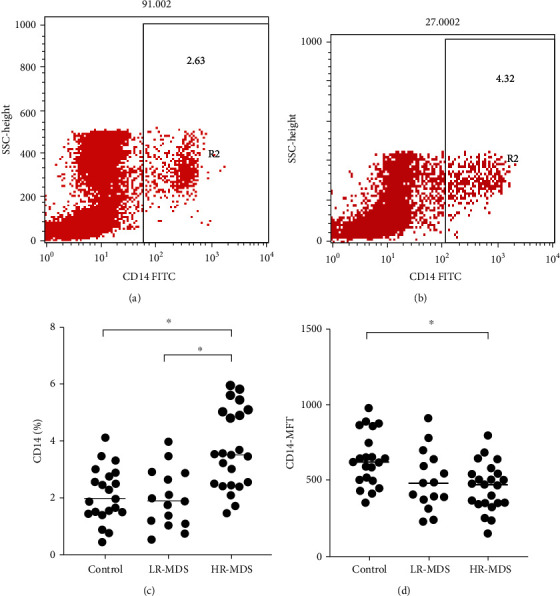
The quantity of monocytes in bone marrow of patients with MDS. (a) Representative dot plots from flow cytometric (FACS) analyses showing the CD14^+^ cell frequency among bone marrow mononuclear cells obtained from healthy controls. (b) Representative dot plots from FACS analyses showing the CD14^+^ cell frequency among bone marrow mononuclear cells obtained from MDS patients. (c) The proportion of CD14^+^ cells from bone marrow of MDS patients and controls. (d) The median fluorescence intensity (MFI) of CD14^+^ cells from bone marrow of MDS patients and controls. ^∗^*p* < 0.05.

The proportion of bone marrow monocytes was 2.11 ± 0.93% in the control group, 1.96 ± 1.53% in the LR-MDS group, and 3.66 ± 3.38% in the HR-MDS group. There was no significant difference in the proportion of bone marrow monocytes between the normal control group and the LR group, and the proportion of bone marrow monocytes was significantly higher in the HR group than in the control group (*p* < 0.05). The proportion of bone marrow monocytes in the HR group was higher than that in the LR group, and the difference was statistically significant (*p* < 0.05; Figure 1).

The median fluorescence intensity (MFI) of CD14^+^ cells from the bone marrow in the control group, LR-MDS group, and HR-MDS group was 639.05 ± 359.78, 501.43 ± 374.44, and 458.26 ± 306.72, respectively. There was no significant difference in the MFI of CD14^+^ cells between the normal control group and the LR group, and the MFI of CD14^+^ cells was significantly lower in the HR group than in the control group (*p* < 0.05). The difference between the LR and HR groups was not statistically significant (Figure 1).

### 3.2. The Number of M2 Macrophages Was Increased in the Bone Marrow of HR-MDS Patients

The proportion of M1 macrophages in the bone marrow monocytes was 6.41 ± 7.09% in the control group, 8.08 ± 10.31% in the LR-MDS group, and 7.80 ± 9.41% in the HR-MDS group. There were no statistically significant differences among the three groups.

The proportion of M2 macrophages in the bone marrow monocyte was 1.82 ± 2.47% in the control group, 3.18 ± 3.79% in the LR-MDS group, and 3.93 ± 3.81% in the HR-MDS group. The proportion in the HR-MDS group was significantly higher than that in the control group, and the difference was statistically significant (*p* < 0.05).

**Figure 2:**
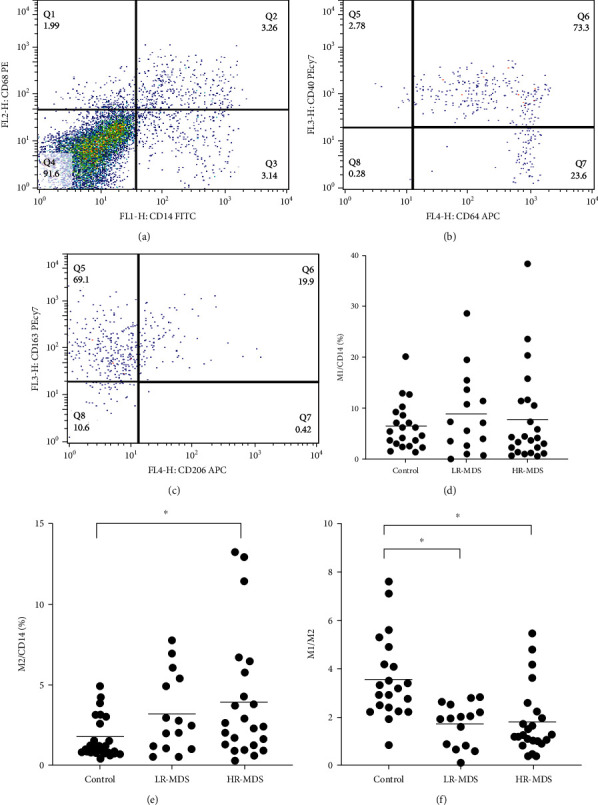
The percentage of macrophages in bone marrow of patients with MDS. (a) Representative dot plots from flow cytometric (FACS) analyses showing the macrophage (CD14^+^CD68^+^ cells) frequency among bone marrow mononuclear cells. (b) Representative dot plots from FACS analyses showing the M1 macrophage (CD64^+^CD40^+^ macrophages) frequency among bone marrow mononuclear cells. (c) Representative dot plots from FACS analyses showing the M2 macrophage (CD206^+^CD163^+^ macrophages) frequency among bone marrow mononuclear cells. (d) The frequency of M1 macrophages from bone marrow of MDS patients and controls. (e) The frequency of M2 macrophages from bone marrow of MDS patients and controls. (f) The ratio of M1/M2 macrophages from bone marrow of MDS patients and controls. ^∗^*p* < 0.05.

The ratio of M1 to M2 macrophages was 3.50 ± 3.22 in the control group, 1.68 ± 0.78 in the LR-MDS group, and 1.80 ± 0.88 in the HR-MDS group. The ratio of M1 to M2 macrophages in the control group was significantly higher than that in the LR-MDS and HR-MDS groups (*p* < 0.05). There was no significant difference in the ratio of M1 to M2 macrophages between the HR-MDS and LR-MDS groups (Figure 2).

### 3.3. The Expression of IL-1*β* and TNF-Alpha mRNA of M1 Macrophages *In Vitro* Was Decreased

The level of IL-1*β* mRNA was 2.07 ± 1.66 in the control group, 0.5 ± 0.6 in the LR-MDS group, and 0.98 ± 0.72 in the HR-MDS group. Compared with the control group, the expressions of IL-1*β* mRNA in the LR-MDS and the HR-MDS groups were lower, and the difference was statistically significant (*p* < 0.05), while the difference between the LR-MDS and HR-MDS groups has no statistical significance.

**Figure 3:**
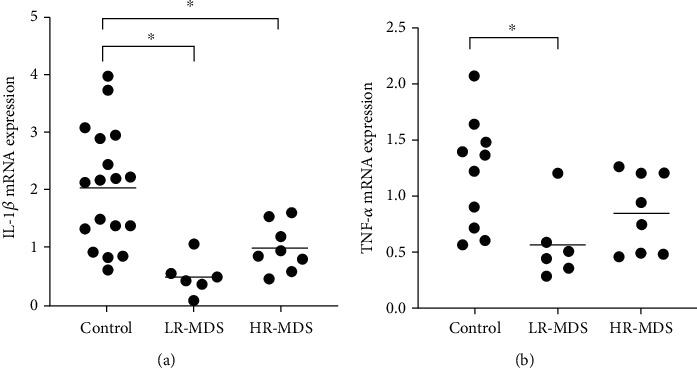
The expression of IL-1*β* and TNF-*α* mRNA in M1 macrophages in vitro. (a) The expression of IL-1*β* mRNA in M1 macrophages. (b) The expression of TNF-*α* mRNA in M1 macrophages. ^∗^*p* < 0.05.

The level of TNF-alpha mRNA was 1.20 ± 0.75 in the control group, 0.55 ± 0.33 in the LR-MDS group, and 0.85 ± 0.36 in the HR-MDS group. Compared with the control group, the expressions of TNF-alpha mRNA in the LR-MDS and HR-MDS groups were lower, and the differences were statistically significant (*p* < 0.05) (Figure 3).

## 4. Discussion

Bone marrow macrophages play an important role in maintaining the homeostasis of the hematopoietic stem cell niche. Removing macrophages can release hematopoietic stem cells into the peripheral blood [8]. CD14^+^ monocytes/macrophages could increase the expansion of erythroid progenitor cells and increase the number of CD34^+^ HSPCs through coculture [9].We previously found that the proportion of peripheral blood monocytes in patients with MDS increased, and the phagocytic ability of differentiated macrophages decreased [7]. In the present study, we found that the proportion of monocytes in the bone marrow of patients with HR-MDS was significantly higher than that of the control group, and the MFI of cell surface antigen CD14 was also significantly different from that observed in the control group. As the disease progressed, the number of abnormal monocytes increased in the BM of the patients. Monocytes showed abnormal maturation and differentiation.

Tissue macrophages and inflammatory macrophages are derived from monocytes in the peripheral blood or from the embryonic origin of tissue macrophages, which have strong plasticity [10]. To adapt to changes in the microenvironment, macrophages can polarize into different types [11]. The functions, cytokines, and surface markers of polarized macrophages are different. Macrophage polarization is generally divided into two categories: classical polarization of type I macrophages (M1) and alternative polarization of type II macrophages (M2) [12]. Studies have found that M1 macrophages are usually induced by IFN-*γ*, LPS, and toll-like receptor agonists. These macrophages secrete proinflammatory factors such as IL-6, IL-12, IL-1*β*, and TNF-*α* and highly express MHC class I and MHC class II molecules that recognize tumor-specific antigens. Therefore, M1 macrophages play important roles in the inflammatory response and antitumor immune response. In contrast, M2 macrophages play important roles in anti-inflammatory activity and tumor growth. M2 macrophages are further divided into four subtypes: M2A, M2B, M2C, and M2D [6]. Studies have shown that tumor-associated macrophages (TAMs) are similar to M2 macrophages, and the M2D subtype is considered to be tumor-associated macrophages [13]. Sica and Mantovani [6] found that the phenotype of TAM macrophages was M2, for example, the IL-12^low^ IL-10^high^ in an advanced stage of tumors. Other researchers believe that such macrophages are conducive to tumor growth, survival, and angiogenesis [6, 14–16].

In this study, we compared the proportion of macrophages, the ratio of M1 to M2, and the expression of macrophage surface molecules between patients with MDS and the control group. We found that the ratio of M2 macrophages to monocytes was higher in patients with MDS. The ratio of M1 to M2 macrophages was lower in the MDS group. There was no significant difference in the proportion of M1 macrophages between MDS patients and the control group. The results showed that with the development of MDS, the macrophages in the bone marrow further polarized to the M2 subtype and not to the M1 subtype, and the antitumor effect of macrophages was insufficient.

In this study, we found that the expression of IL-1*β* and TNF-*α* mRNA in M1 macrophages of patients with MDS was significantly lower than that in the control. Dumont et al. [17] found that macrophages stimulated by LPS highly expressed IL-1*β* and TNF-*α* and inhibited the proliferation of colon cancer cells. Klimp et al. [18] also confirmed that macrophages stimulated by LPS and IFN-*γ* could kill tumor cells by secreting TNF. Studies have shown that TNF-*α* promotes the apoptosis of MDS progenitor cells [19], and the concentration of TNF-*α* in the bone marrow supernatant and plasma of MDS patients was increased, and the expression of TNF receptor and TNF-*α* mRNA was increased in mononuclear cells of MDS. As a proinflammatory factor, IL-1*β* has various effects on hematopoiesis. IL-1*β* at physiological concentrations can promote the secretion of GM-CSF and other colony-stimulating factors and promote hematopoiesis [20]. Allampallam et al. [21] found that the mononuclear cells of MDS also expressed IL-1*β*. Basiorka et al. [22] found that MDS HSPC overexpressed inflammatory protein and activated the NLRP3 complex, thus activating cysteinase 1, secreting IL-1*β*, and promoting cell death. Therefore, we found that the expression of IL-1*β* and TNF-*α* mRNA decreased by culturing macrophages from MDS patients *in vitro* and stimulating them to differentiate into M1 using LPS and IFN-*γ* treatment. We speculated that the inflammatory factors secreted by M1 macrophages in the MDS group were decreased, and M1 macrophages in patients with MDS had insufficient antitumor function, and their proinflammatory and antitumor effects were weakened, which may be related to the occurrence and progression of MDS.

The increase in M2 polarization in the bone marrow of patients with MDS is beneficial for the proliferation of MDS clonal cells. Repolarization of M2 cells to the M1 phenotype is a method of cancer immunotherapy, which can effectively restore the response of the innate and adaptive immune systems, leading to tumor regression [23]. Demethylation drugs, decitabine and azacytidine, are the standard treatments for relatively high-risk MDS. Demethylation drugs combined with histone deacetylase inhibitors or PD1/PDL1 could increase M1 macrophages and activate type I interferon [24, 25]. Therefore, using a combination of drugs that can promote M1 polarization may be an interesting direction for the treatment of MDS.

Our study has some limitations, such as whether the induced M1 macrophages express the surface markers of M1 cells, such as iNOS and STAT-1, and the levels of TNF-*α* and IL-1*β* secreted by these M1 macrophages.

In conclusion, we found that the polarization of bone marrow macrophages in patients with MDS was abnormal, M1 macrophages were relatively reduced, and IL-1*β* and TNF were decreased. This may be a manifestation of an abnormal bone marrow microenvironment in patients with MDS. Regulation of macrophage polarization may be one of the directions of MDS targeted therapy.

## Figures and Tables

**Table 1:** The characteristics of myelodysplastic syndrome patients.

Case	Sex	Age	Diagnosis	Cytogenetics	IPSS
1	Male	21	RARS	46,XY	Low
2	Male	63	RCMD	46,XY	Low
3	Female	38	RAEB2	46,XY	Int-2
4	Male	46	RCMD	46,XY,-2,-12,+mar,19+,9P+	Int-2
5	Female	57	RAEB2	46,XX	Int-2
6	Male	58	RAEB2	46,XY	Int-2
7	Male	59	RAEB2	46,XY	Int-2
8	Male	59	RAEB2	46,XY	Int-2
9	Female	59	RAEB1	46,XY,13q+	Int-2
10	Male	62	RAEB2	46,XY	Int-2
11	Female	64	RAEB2	46,XX	Int-2
12	Male	65	RCMD	46,XY,del17q31	Int-2
13	Male	67	RAEB2	46,XY	Int-2
14	Female	69	RAEB2	46,XX	Int-2
15	Female	70	RAEB2	46,XX	Int-2
16	Male	76	RAEB2	No result	Int-2
17	Female	79	RAEB2	46,XX	Int-2
18	Male	42	RARS	46,XY,del20q11	Int-1
19	Female	47	RARS	46,XX	Int-1
20	Female	49	RARS	46,XX	Int-1
21	Male	50	RAEB1	46,XX	Int-1
22	Male	50	RCMD	47,XY,+8/46,XY	Int-1
23	Female	51	RAEB1	46,XX	Int-1
24	Male	57	RAEB1	46,XY	Int-1
25	Male	58	RAEB1	46,XY	Int-1
26	Female	62	5q-	5q-	Int-1
27	Male	62	RA	46,XY	Int-1
28	Female	64	RAEB1	46,XX	Int-1
29	Female	74	RARS	46,XX	Int-1
30	Female	74	RCMD	46,XX	Int-1
31	Male	27	RAEB2	3p+,-18,+mar	High
32	Female	29	RAEB2	20q-,5q-,7q-	High
33	Male	30	RAEB2	47,XY,+8/46,XY	High
34	Male	60	RAEB2	45,XY,-7	High
35	Male	68	RAEB2	46,XY,+8/45,XY+8,-6,-7	High
36	Female	76	RAEB2	del5q33,del5q31,del7q311,del7q3	High
37	Female	77	RAEB2	45,XX,-5,-2,45,XX,+mar,-5,3P-	High
38	Female	79	RAEB2	45,XX,-7	High

## Data Availability

The data used to support the findings of this study are included within the article.

## Supplementary Materials

Supplementary Materials Supplemental Figure 1: the gating strategy for macrophages. (A) Bone marrow mononucleated cells were gated with SSC and FSC. (B) Monocytes were gated with CD14. (C) Macrophages were defined with CD14^+^CD68^+^ cells. (D) M1 macrophages were defined with CD14^+^CD68^+^CD40^+^CD64^+^ cells. (E) M2 macrophages were defined with CD14^+^CD68^+^CD163^+^CD206^+^ cells.
