# Supplementary figures and images for: Abnormal Macrophage Polarization in Patients with Myelodysplastic Syndrome
Source: Mediators Inflamm. 2021 Jul 9;2021:9913382. doi: 10.1155/2021/9913382 (PMC8286189; doi:10.1155/2021/9913382)

Supplementary


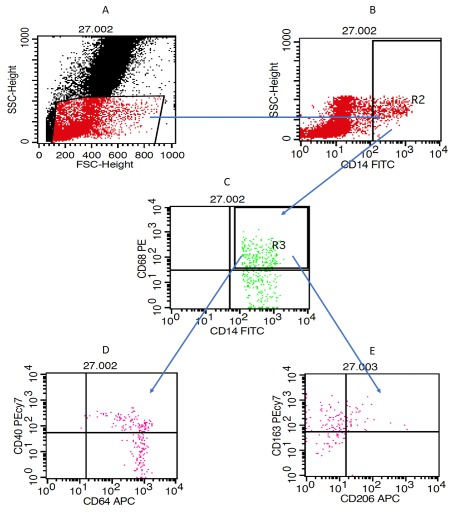

Supplement: Supplementary Materials — Supplemental Figure 1: the gating strategy for macrophages. (A) Bone marrow mononucleated cells were gated with SSC and FSC. (B) Monocytes were gated with CD14. (C) Macrophages were defined with CD14+CD68+ cells. (D) M1 macrophages were defined with CD14+CD68+CD40+CD64+ cells. (E) M2 macrophages were defined with CD14+CD68+CD163+CD206+ cells. [file 9913382.f1.docx]
